# Efficacy of radioiodine therapy for treating 20 patients with pulmonary metastases from differentiated thyroid cancer and a meta-analysis of the current literature

**DOI:** 10.1007/s12094-017-1792-1

**Published:** 2017-11-08

**Authors:** X. Zhang, D.-S. Liu, Z.-S. Luan, F. Zhang, X.-H. Liu, W. Zhou, S.-F. Zhong, H. Lai

**Affiliations:** 1 Department of Traditional Chinese Medicine, Qilu Hospital of Shandong University, Jinan, China; 2 Department of Nuclear Medicine, The 88th Hospital of PLA, Tai’an, China; 3 Department of Endocrinology, Qilu Hospital of Shandong University, 107 Wenhua Xi Road, Jinan, 250012 People’s Republic of China

**Keywords:** Differentiated thyroid cancer, Pulmonary metastasis, Radioiodine therapy, Efficacy, Meta-analysis

## Abstract

**Background:**

Radioiodine therapy (RAI) after total or near-total thyroidectomy is a recommended treatment for patients with pulmonary metastasis from differentiated thyroid cancer (DTC). However, the total effective rate of iodine-131 therapy remains controversial. This study aimed to determine the efficacy of RAI for treating patients with pulmonary metastasis from DTC, and to identify independent predictors of its efficacy.

**Methods:**

We conducted a retrospective study to evaluate 20 patients with pulmonary metastasis from DTC who underwent RAI at our center at first and performed a meta-analysis to evaluate relevant literature regarding the overall efficacy of RAI and subgroup-specific efficacies subsequently.

**Results:**

The efficacy rate at our center was 40%, and no significant differences were observed according to sex, age, pathological type, metastasis state, or interval between the initial RAI and final surgery. The meta-analysis revealed that the pooled overall efficacy rate was 58%, and significant differences were observed when we compared pulmonary metastasis versus pulmonary and other distant metastasis, age of < 40 years versus age of ≥ 40 years, papillary thyroid cancer versus follicular thyroid cancer and male patients versus female patients.

**Conclusions:**

These results suggest that RAI is an effective treatment for patients with pulmonary metastasis from DTC after surgical treatment. The efficacy of RAI was significantly predicted by the presence of papillary thyroid cancer, age of < 40 years, the absence of non-lung distant metastasis and female patients.

## Introduction

Differentiated thyroid cancer (DTC) accounts for the majority of thyroid cancers, and includes papillary thyroid cancer (PTC) and follicular thyroid cancer (FTC) [1]. The lungs are the most common site of distant metastasis in patients with DTC [2–4]. Based on the 2009 guidelines from the American Thyroid Association, total or subtotal thyroidectomy followed by radioiodine therapy (RAI) is recommended for patients with pulmonary metastasis from DTC [5], and most studies regarding this treatment have revealed significant efficacy [6–9]. However, the overall efficacy of iodine-131 (^131^I) therapy remains controversial. On the one hand, it is reported that the efficacy of RAI to DTC is associated with the expression of sodium iodide symporter (NIS). The presentation of NIS in DTC cells is responsible for radioiodine uptake. Some differentiated thyroid cancers have a poor prognosis for the iodine uptake is impaired [10]. On the other hand, most studies have used a retrospective design, did not use randomization, or did not control for the limitations of ethical principles and medical reality. Therefore, the present study retrospectively evaluated the clinicopathological characteristics and responses to ^131^I therapy among 20 patients with pulmonary metastasis from DTC. Furthermore, we performed a meta-analysis of the current literature to identify factors that independently predicted RAI efficacy in cases of pulmonary metastasis from DTC.

## Methods

### Patients

Between 2005 and 2014, 20 patients with pulmonary metastases in DTC underwent RAI after undergoing surgical treatment at our Department of Nuclear Medicine (The 88th PLA Hospital). All the patients included in our retrospective study were consisted of 10 male patients and 10 female patients. The ages at the diagnosis ranged from 19 to 76 years (mean 45.7 ± 17.6 years), and 5 patients (25%) experienced distant metastases to the bone. The pulmonary and bone metastases were mainly diagnosed based on pre- or post-treatment uptake of radioiodine that was identified during whole-body scanning (WBS). In cases where the WBS did not detect pulmonary metastasis, we also used computed tomography to screen for pulmonary nodes and tested for elevated serum thyroglobulin (Tg) levels.

### Treatment

The RAI was administered as a single 30–215 mCi dose of ^131^I, with an intertreatment interval of 3–6 months. All patients received no more than five treatments, and the results were evaluated using WBS and Tg measurements. At 3–5 days after each treatment, a quantitative WBS was performed using a dual-head gamma camera (GE Hawkeye SPECT or Symbia T2). Serum Tg measurements were performed using a chemiluminescence assay before each oral treatment.

### Criteria to evaluate treatment efficacy

Complete remission (CR) was defined as a serum Tg level of ≤ 1.5 ng/mL and no clear ^131^I uptake was detected during the WBS. Partial remission (PR) was defined as a serum Tg level of ≤ 1.5 ng/mL and no clear ^131^I uptake was detected during the WBS. The treatment was considered effective in cases with CR or PR. Treatment ineffectiveness was defined as a serum Tg level of > 1.5 ng/mL and a clear ^131^I uptake was detected during the WBS.

### Statistical analysis

Continuous data were presented as mean ± standard deviation, and categorical data were presented as number (%). Differences in treatment response were compared using Fisher’s exact test and IBM SPSS software (version 21.0; IBM Corp., Armonk, NY, USA). Differences were considered significant at *P* values of < 0.05.

### Meta-analysis

We also performed a meta-analysis to evaluate the overall efficacy of RAI for patients with pulmonary metastasis from DTC, and to identify independent predictors of its efficacy. A search of the PubMed database was performed in October 2016 by two independent investigators using the following terms: “thyroid carcinoma OR thyroid neoplasm OR thyroid neoplasms OR thyroid cancer” AND” iodine OR radioiodine “AND” lung metastas* OR pulmonary metastas*”. The inclusion criteria were: (1) the report was published in English (2) the study evaluated RAI for patients with pulmonary metastasis from DTC, and (3) the patients all underwent total or subtotal thyroidectomy before the RAI. The exclusion criteria were: (1) reviews, (2) studies with a small sample size (< 15 patients), and (3) unclear information regarding the outcomes and efficacy of RAI.

The meta-analysis was performed using the Cochrane Collaboration Review Manager (RevMan version 5.3). Pooled estimates of the effective rates were calculated using either a fixed-effects or random-effects model based on heterogeneity. Significant heterogeneity was considered present based on a Chi-square test *P* value of ≤ 0.05 or an *I* ^2^ value of > 50% [11]. The overall efficacy results were expressed as risk difference (RD) with the 95% confidence interval (CI), and the results of the subgroup analyses were expressed as odds ratios (ORs) with 95% CIs. A funnel plot was used to test for the presence of publication bias.

## Results

### Results of the RAI at our center

The clinical characteristics of the 20 patients from our center are shown in Table 1. The patients received single doses of 30–215 mCi and total doses of 249–895 mCi (mean 559.0 ± 143.1 mCi) during 2–5 treatments (mean 4.5 ± 0.9 treatments). We observed CR in 5 patients (25%) and PR in 3 patients (15%), and the RAI was considered ineffective for the remaining 12 patients (60%). Thus, the efficacy rate at our center was 40%. We did not detect any statistically significant differences in efficacy according to sex, age, pathological type, metastasis state, or interval between the initial RAI and the final surgery.

**Table 1:** Clinical feature of the 20 patients with pulmonary metastases from DTC

Clinical feature	No. of patients (%)	Effectiveness (CR/PR)	Ineffectiveness	*P*
Gender				
Male	10 (50.0%)	3	7	0.650
Female	10 (50.0%)	5	5	
Age (years)				
< 40	9 (45.0%)	5	4	0.362
≥ 40	11 (55.0%)	3	8	
Pathological type				
PTC	14 (70.0%)	7	7	0.325
FTC	6 (30.0%)	1	5	
Metastasis state				
PM	5 (25.0%)	0	5	0.055
PBM	15 (75.0%)	8	7	
Interval between initial RAI and final surgery, months				
< 5	7 (35.0%)	1	6	0.158
≥ 5	13 (65.0%)	7	6	

*PM* pulmonary metastases, *PBM* pulmonary and bone metastases

### Results of the meta-analysis

**Fig. 1:**
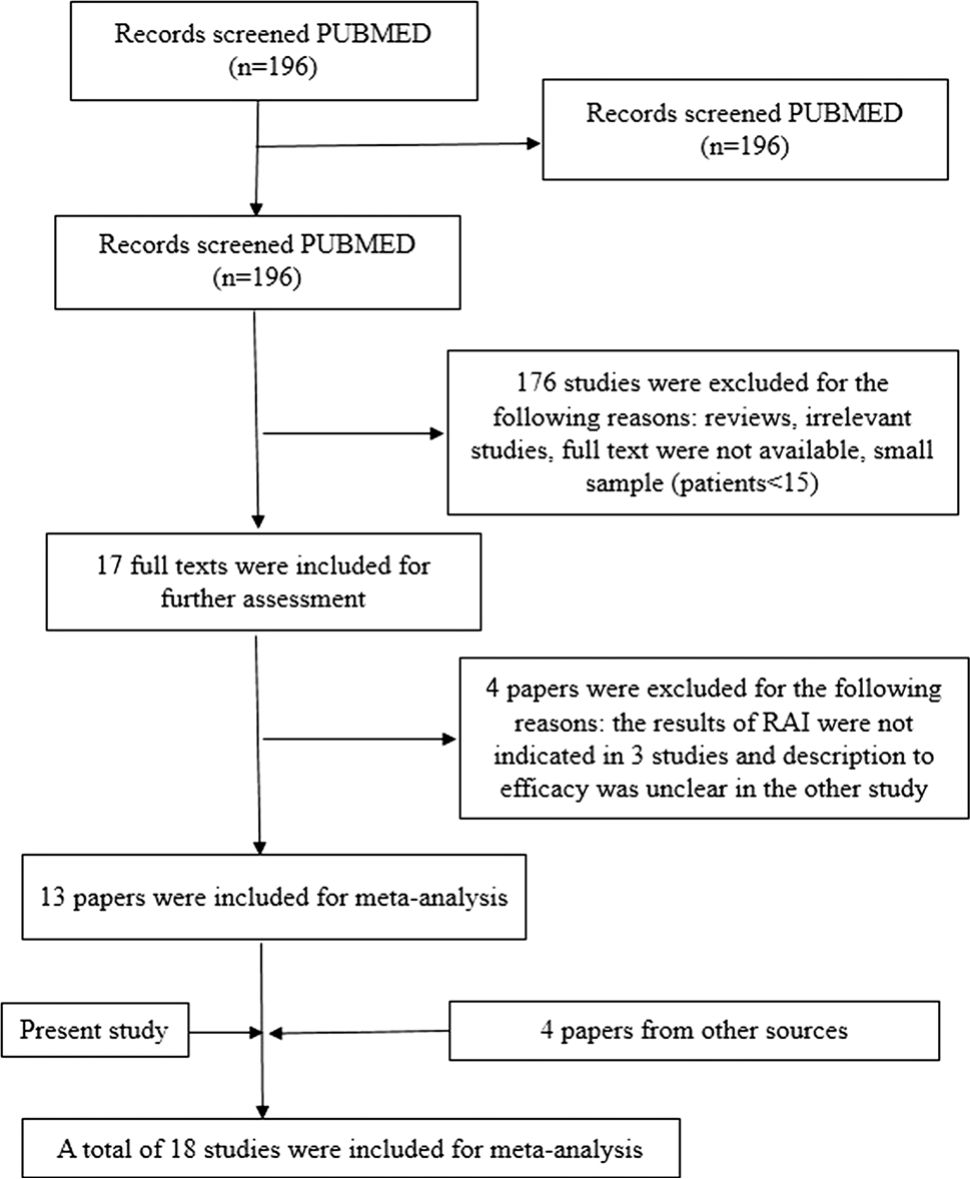
The article selection procedure

Figure 1 shows that we identified 196 potentially relevant reports, although only 13 reports were included after applying the inclusion and exclusion criteria. We also identified 4 reports from other sources that fulfilled the inclusion criteria. In addition to CR and PR, some studies defined effectiveness using cases with no evidence of disease, stable disease, or being alive with disease. The overall efficacy of RAI for pulmonary metastasis from DTC varied from 37.5 to 96.8% (Table 2). When we combined our study with the previous studies, we identified 18 studies with 1228 patients.

**Table 2:** Characteristics of included studies

Study	Definition of effectiveness	Total effective rate (*n*)
Bal et al. [6]	Ablated or partially ablated	90.0% (18/20)
Biko et al. [17]	Complete biochemical remission or stable partial remission	80.0% (16/20)
Durante et al. [3]	No radiological abnormalities or No persistent radioiodine uptake	62.3% (190/305)
Hindié et al. [18]	Apparent remission or persistent disease or recurrent remission and relapse	80.0% (16/20)
Ilgan et al. [13]	Complete response or stable disease	40.5% (17/42)
Kalender et al. [12]	Complete response or partial response	47.1% (8/17)
Küçük et al. [14]	Tg < 30 ng/mL	37.5% (12/32)
Lee et al. [19]	Alive with disease or no evidence of disease	70.0% (14/20)
Long et al. [7]	Complete response or partial response	95.2% (20/21)
Dottorini et al. [23]	^131^I lung uptake was no longer evident on the WBS and Tg continued to decrease	75.0% (12/16)
Pace et al. [20]	Complete remission or partial remission or stable disease	72.2% (13/18)
Pitoia et al. [15]	No evidence of disease or biochemical persistence	45.8% (11/24)
Present study	Complete remission or partial remission	40.0% (8/20)
Samuel et al. [8]	Complete response or partial response	96.2% (25/26)
Schlumberger et al. [21]	Complete remission	87.0% (20/23)
Schlumberger et al. [16]	Remission	39.2% (112/286)
Song et al. [22]	Effective or stable	82.4% (211/256)
Tachi et al. [9]	Partial remission or no change	96.8% (60/62)

**Fig. 2:**
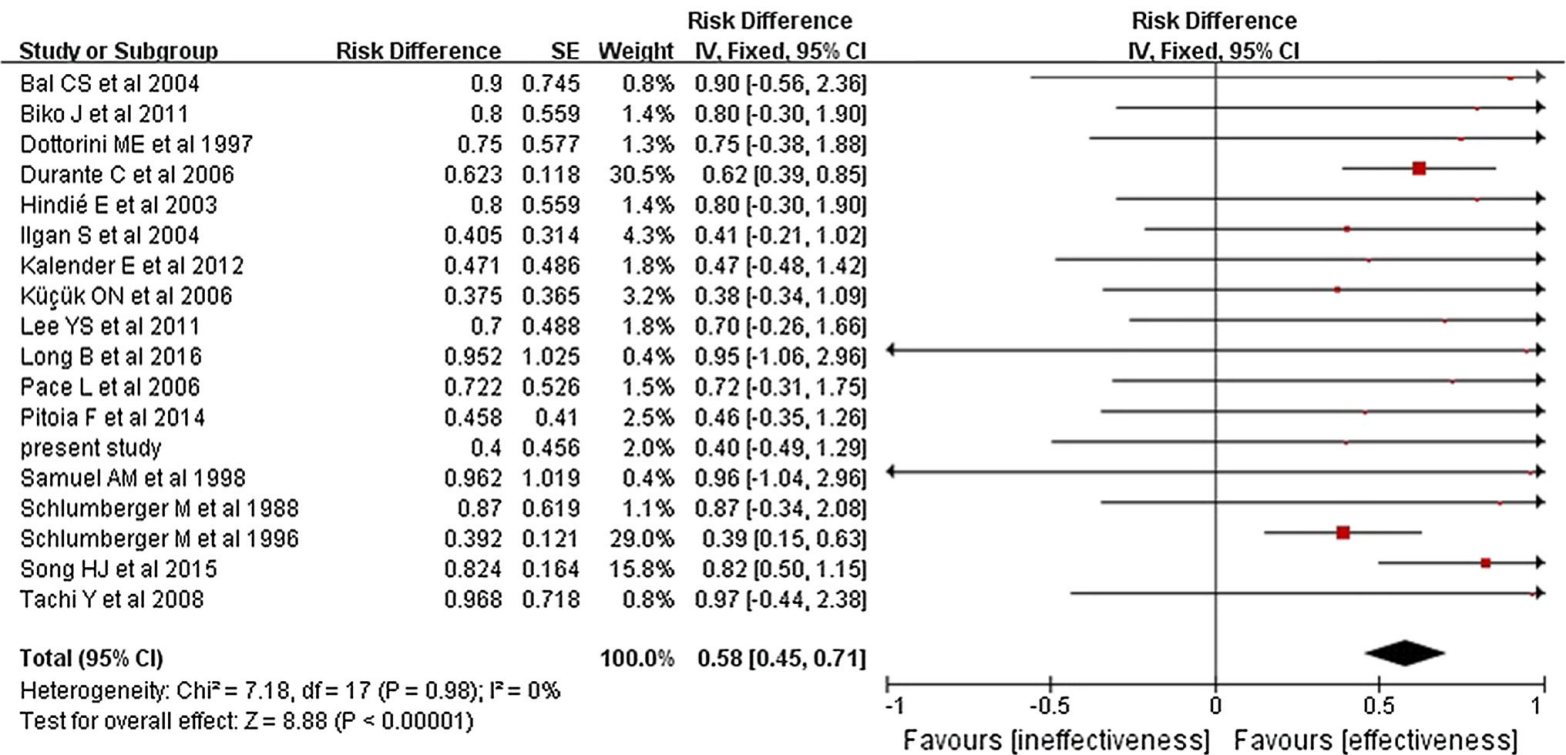
Forest plots for the pooled overall efficacy rate using a fixed-effects model

**Fig. 3:**
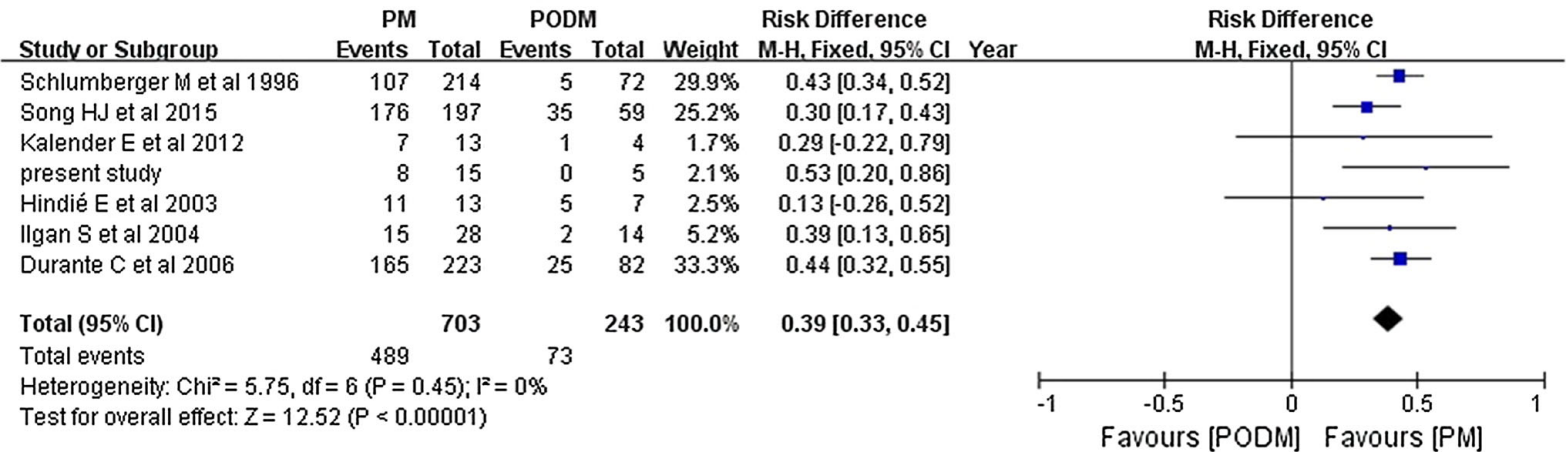
Forest plots comparing the efficacy rates in the PM and PODM groups using a fixed-effects model. *PM* pulmonary metastasis, *PODM* pulmonary and other distant metastasis

**Fig. 4:**
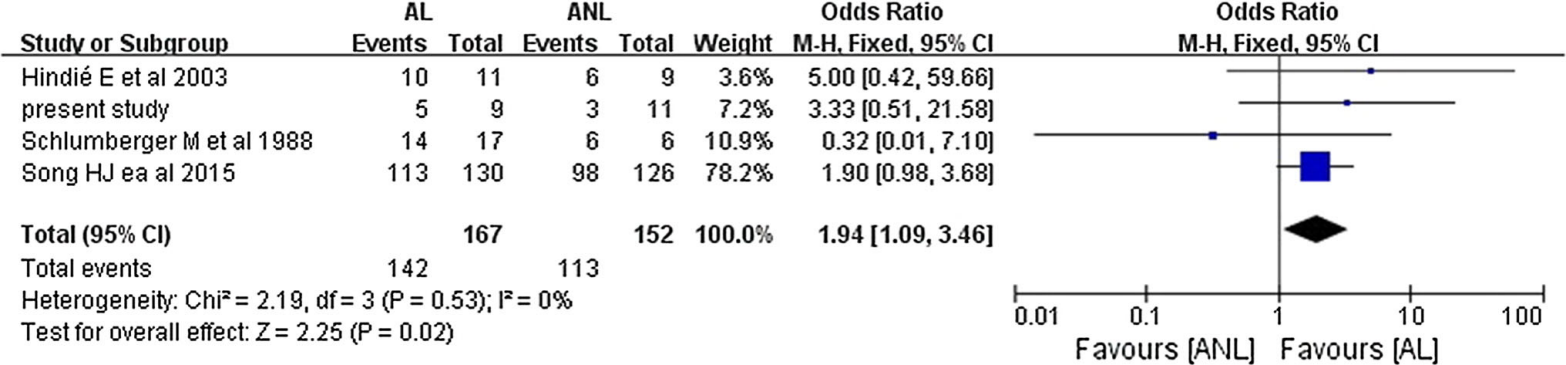
Forest plots comparing the efficacy rates in the AL and ANL groups using a fixed-effects model. *AL* age of < 40 years, *ANL* age of ≥ 40 years

**Fig. 5:**
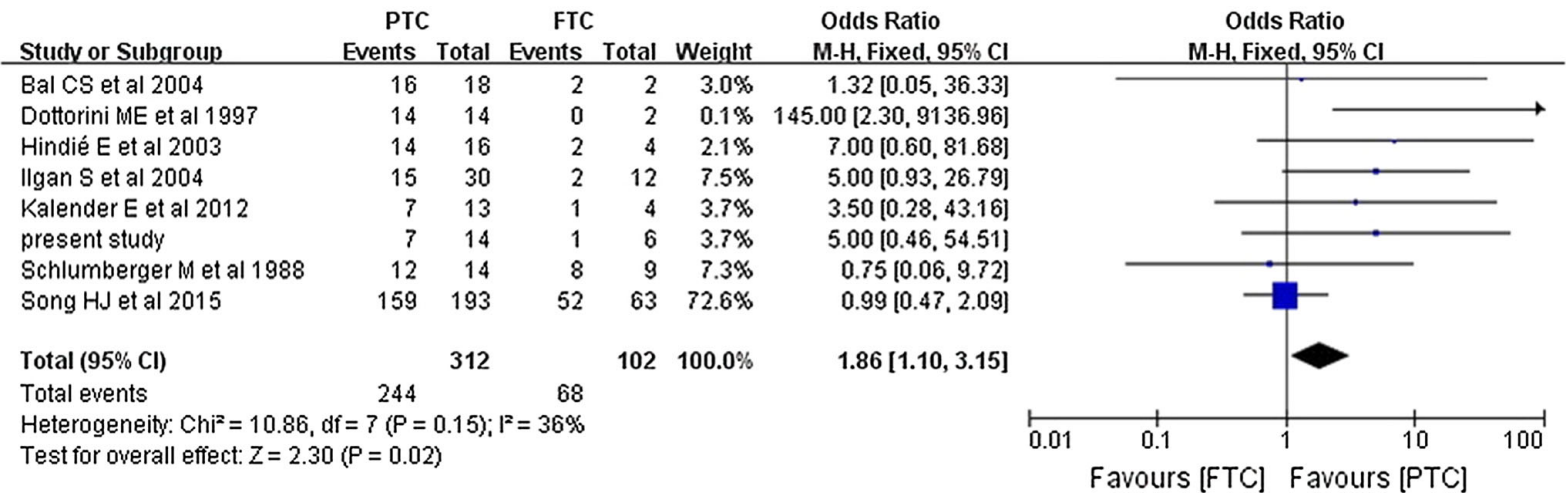
Forest plots comparing the efficacy rates in the PTC and FTC groups using a fixed-effects model. *PTC* papillary thyroid cancer, *FTC* follicular thyroid cancer

**Fig. 6:**
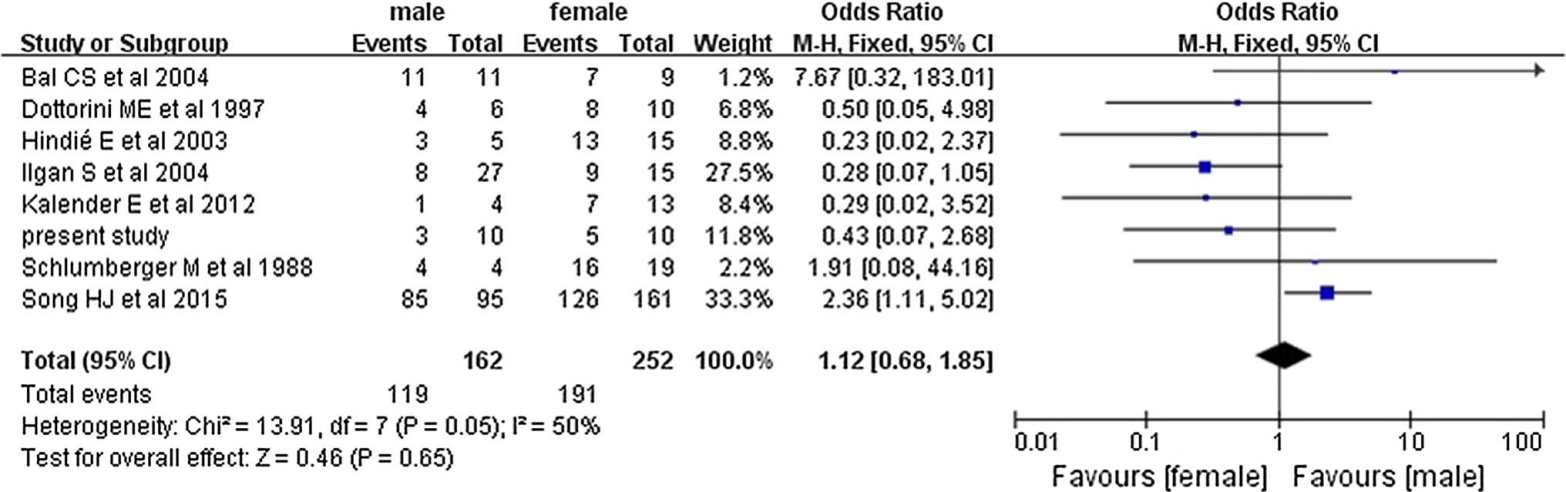
Forest plots comparing the efficacy rates among men and women using a fixed-effects model

The pooled overall efficacy rate was 58% (RD 0.58, 95% CI 0.45–0.71, *P* < 0.00001) (Fig. 2). Subgroup analyses revealed significant differences in the total efficacy rates when we compared pulmonary metastasis versus pulmonary and other distant metastasis (OR 7.29, 95% CI 5.03–10.54, *P* < 0.00001) (Fig. 3), age of < 40 years versus age of ≥ 40 years (OR 1.94, 95% CI 1.09–3.46, *P* = 0.02) (Fig. 4), and PTC versus FTC (OR 1.86, 95% CI 1.10–3.15, *P* = 0.02) (Fig. 5). No significant difference was observed when we compared the male and female patients (OR 1.12, 95% CI 0.68–1.85, *P* = 0.65) (Fig. 6).

**Fig. 7:**
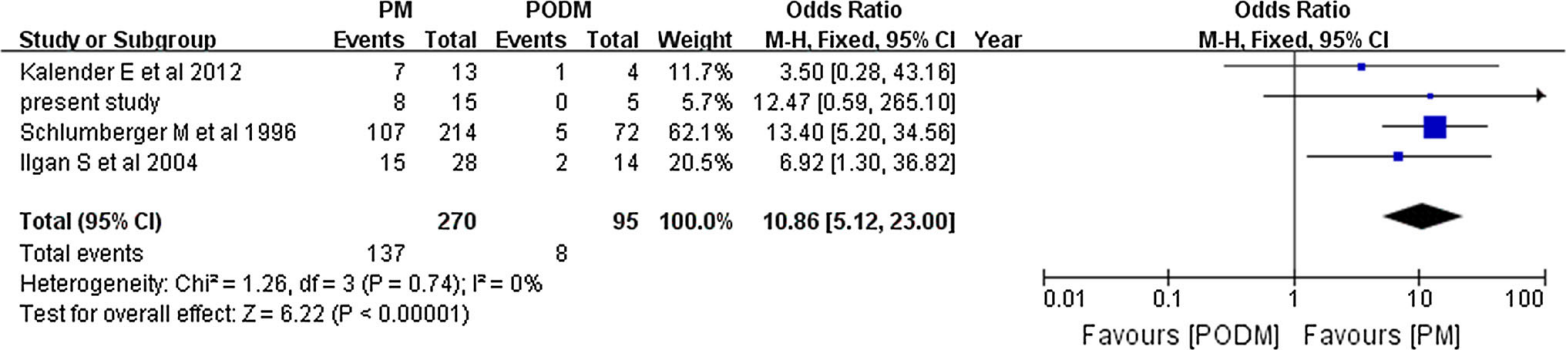
Forest plots comparing the efficacy rates which correlated most closely with ours in the PM and PODM groups using a fixed-effects model. *PM* pulmonary metastasis, *PODM* pulmonary and other distant metastasis

**Fig. 8:**
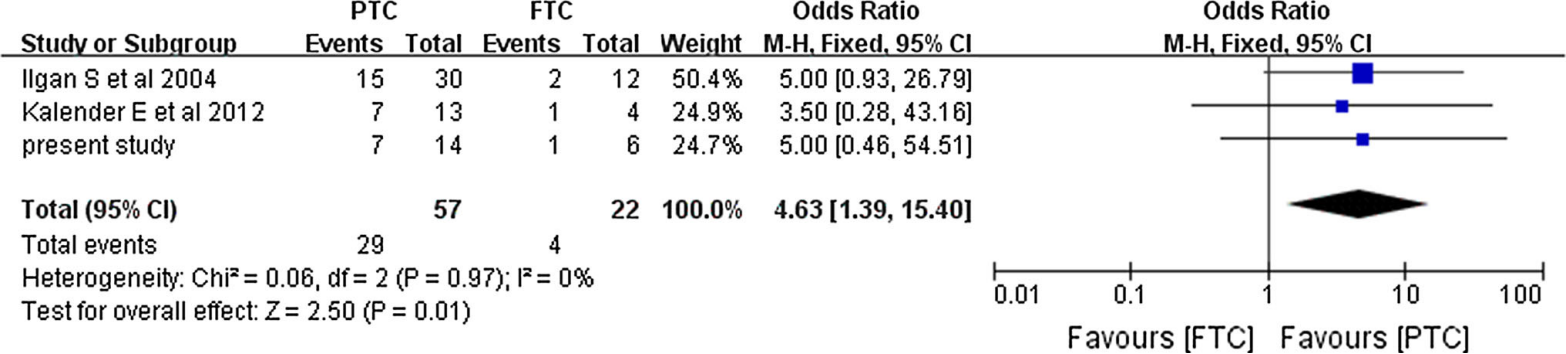
Forest plots comparing the efficacy rates which correlated most closely with ours in the PTC and FTC groups using a fixed-effects model. *PTC* papillary thyroid cancer, *FTC* follicular thyroid cancer

**Fig. 9:**
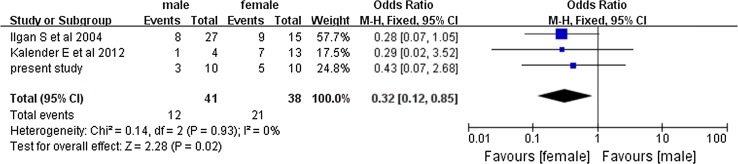
Forest plots comparing the efficacy rates which correlated most closely to ours among men and women using a fixed-effects model

We made a comparison between the results which correlated most closely to the results produced by us to give a better insight into the variation (with a total efficacy rate at 35–50%). Significant differences were revealed in the total efficacy rate not only when we compared pulmonary metastasis versus pulmonary and other distant metastasis (OR 10.86, 95% CI 5.12–23.00, *P* < 0.00001) (Fig. 7) and PTC versus FTC (OR 4.63, 95% CI 1.39–15.4, *P* = 0.01) (Fig. 8), but also when we compared the male and female patients (OR 0.32, 95% CI 0.12–0.85, *P* = 0.02) (Fig. 9).

**Fig. 10:**
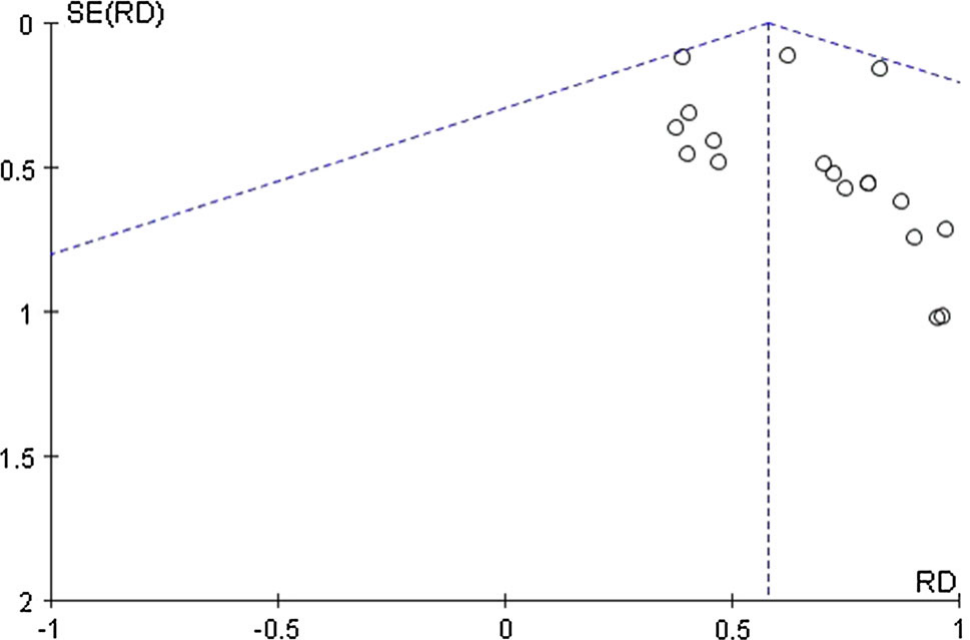
Funnel plot for the included reports. *RD* risk difference, *SE* standard error

Figure 10 shows the funnel plot of the included studies, and does not indicate that significant publication bias is present.

## Discussion

Numerous studies have reported the efficacy of RAI, which plays a key role in the treatment of patients with pulmonary metastasis from DTC. This retrospective study evaluated 20 patients who received RAI for pulmonary metastasis from DTC, and found that the efficacy rate was relatively low (40%). Furthermore, none of the patients’ characteristics were significantly associated with RAI efficacy. Therefore, we also performed a meta-analysis of related studies, and found that the overall efficacy rate was 58%. To our knowledge, this is the first meta-analysis to evaluate the efficacy rate of RAI for pulmonary metastasis from DTC. Based on the results of the meta-analysis, it appears that RAI efficacy is significantly predicted by the presence of PTC, an age of < 40 years, the absence of non-lung distant metastasis and female patients. Further relevant studies needed to interpret differences above.

Similar to our results, some studies have revealed a low efficacy rate for RAI treatment of pulmonary metastasis from DTC [3, 12–16], although other studies have revealed higher efficacy rates [6–9, 17–23]. These differences may be related to the different criteria for dosage selection and efficacy evaluation. Secondly, the small sample size in the present study may be responsible for our inability to identify significant predictors of RAI efficacy. Lastly, the RAI efficacy is associated with the expression of NIS. The impairment of iodine uptake correlated directly with the RAI efficacy of DTC [10].

Older patients at DTC diagnosis often present with more aggressive cancer and have a decreased age adjusted disease-free and thus have worse prognosis [24, 25]. It is speculated that the difference of RAI efficacy between PTC and FTC is associated with the diverse metastatic gene signature for PTC and FTC [26]. Heterogeneity in the metastatic tissue may imply that iodine uptake may differ, which may be responsible for the significant difference of RAI efficacy between pulmonary metastasis patients and pulmonary and other distant metastasis patients [10]. As for gender, it is same to our first subgroup analysis about gender; most of the studies reported that there is no significant difference between male and female patients in RAI efficacy. But when we conducted a comparison between our retrospective study results and the results which correlated most closely with the results produced by us, we revealed the significant difference. Different criteria for evaluating the efficacy of RAI may be responsible for the failure to find the significant difference. We speculated that the difference may be associated with estrogen, but more large sample and multicenter clinical studies which have same criteria for evaluating the efficacy of RAI needed to confirm this new conclusion.

The present study has several limitations. First, the meta-analysis only included retrospective studies, and their findings may have been influenced by the absence of randomization or controlling for the limitations of ethical principles and medical reality. Second, each study used slightly different criteria for evaluating the efficacy of RAI. Finally, only the studies published in English were considered. Thus, after randomized controlled trials have been performed in this field, it may be appropriate to perform another meta-analysis to validate our findings.

RAI can be selected as a palliative treatment or a preoperative procedure for patients with pulmonary and bone metastasis while the radical surgical extirpation (complete bone metastasis surgery) could improve the survival for those patients [27, 28]. External radiotherapy is recommended for bone metastasis which cannot be readily excised surgically [29, 30]. Some patient groups with worse prognosis for the failure to uptake iodine may have a try to molecularly targeted therapy, which can increase NIS expression and radioiodine uptake in cancer cells according to some studies [31–33].

## Conclusions

The present study revealed that RAI was an effective treatment for patients with pulmonary metastasis from DTC after surgical treatment. The efficacy of RAI was significantly predicted by the presence of PTC, age of < 40 years, the absence of non-lung distant metastasis and female patients.
